# Crawling towards a map of the brain

**DOI:** 10.7554/eLife.15438

**Published:** 2016-03-31

**Authors:** Laura Masullo, Marco Tripodi

**Affiliations:** MRC Laboratory of Molecular Biology, Cambridge, United Kingdom; MRC Laboratory of Molecular Biology, Cambridge, United Kingdommtripodi@mrc-lmb.cam.ac.uk

**Keywords:** motor circuits, wiring diagram, central pattern generator, <i>D. melanogaster</i>

## Abstract

State-of-the-art techniques reveal a simple circuit of neurons controls the muscle contractions that allow fruit fly larvae to crawl.

**Related research article** Fushiki A, Zwart MF, Kohsaka H, Fetter RD, Cardona A, Nose A. 2016. A circuit mechanism for the propagation of waves of muscle contraction in *Drosophila*. *eLife* **5**:e13253. doi: 10.7554/eLife.13253

**Image** Neural circuits in individual body segments connect to each other to generate waves of muscle contraction

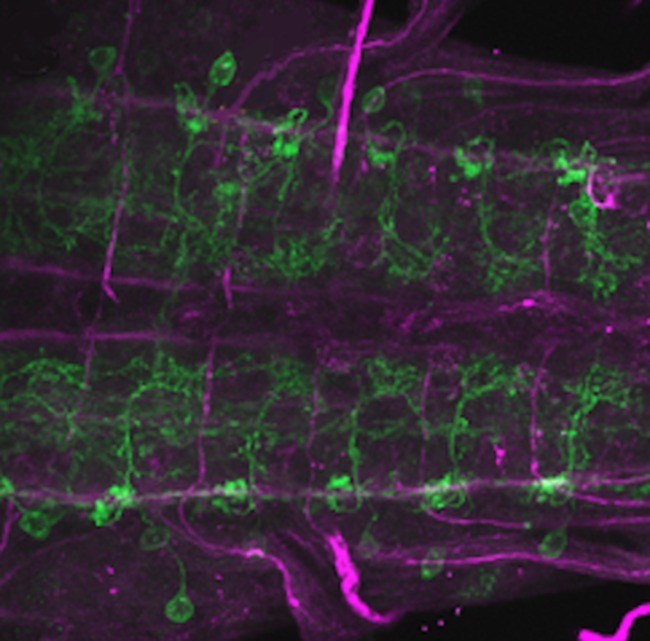

Even the most experienced explorer is better off taking a map with him or her when venturing out into the wild. Similarly, exploring the brain without a map can be a treacherous task. For this reason, neuroscientists have been working for many years to build up maps of how neurons connect to each other. Such maps – which are known as neural “connectomes” – will not on their own explain how the brain processes information, but by imposing constraints on a highly complex system, they help us to understand what is likely, unlikely or impossible.

Now, in eLife, Albert Cardona, Akinao Nose and colleagues – including Akira Fushiki as first author – report how they have combined state-of-the-art electron microscopy with powerful genetic techniques to reveal how the neurons in the ventral nerve cord of *Drosophila* (fruit fly) larvae connect to each other (Fushiki et al., 2016). In doing so, they aimed to identify the neural networks that control crawling movements.

In many animals, patterns of muscle contractions during rhythmic movements – such as walking, swimming or crawling – can happen in the absence of feedback from sensory neurons. The neural networks that control such repetitive patterns are called central pattern generators (Grillner, 2006; Marder et al., 2005; Suster and Bate, 2002).

Crawling requires motor neurons to activate waves of muscle contraction along the body, and the timing of the motor neuron activity in adjacent body segments must be co-ordinated so that when one segment contracts, the segment next to it remains relaxed. Different models can account for this behaviour. One possibility is that there is a chain of nearly independent neural circuits – one in each body segment – that act as oscillators (or clocks) and only weakly influence one another (Cang and Friesen, 2002; Matsushima and Grillner, 1992). Another possibility is that there is a single master clock that triggers the muscles in each segment to contract in turn (Deliagina et al., 1983). Intermediate designs have also been proposed, such as the nearest neighbour-only model, which is composed of neural circuits in chains that are coupled by short distance connections (Gjorgjieva et al., 2013).

So, we have a variety of models that are equally plausible, but how do we figure out which is actually used in nature? Fushiki et al. – who are based at the Janelia Research Campus, the University of Tokyo and Cambridge University – set out to address this question and identified a sub-network that controls the ability of larvae to crawl. They performed their experiments on isolated ventral nerve cords, whereby the central nervous system of the larva is dissected and placed in a recording chamber. The neurons within the isolated ventral nerve cord will continue to produce patterns of activity that recapitulate those present when the animal is crawling. For this reason such a wave of activity is often referred to as “fictive locomotion”.

By using isolated ventral nerve cords, Fushiki et al. identified a sub-network that includes two key neurons repeated in each body segment. The first neuron, termed A27h, acts directly on motor neurons to drive muscle contraction (Figure 1). It also activates the other key neuron – known as a GABAergic dorsolateral neuron (GDL) – in the next segment, which then inhibits the A27h neuron in the corresponding segment. The coordinated activation of A27h and GDL neurons generates the motor commands needed to contract the muscles in one segment while relaxing the muscles in the next. Fushiki et al. show that the two neurons are necessary to ensure that a wave of muscle contraction moves along the body.

**Figure 1.**
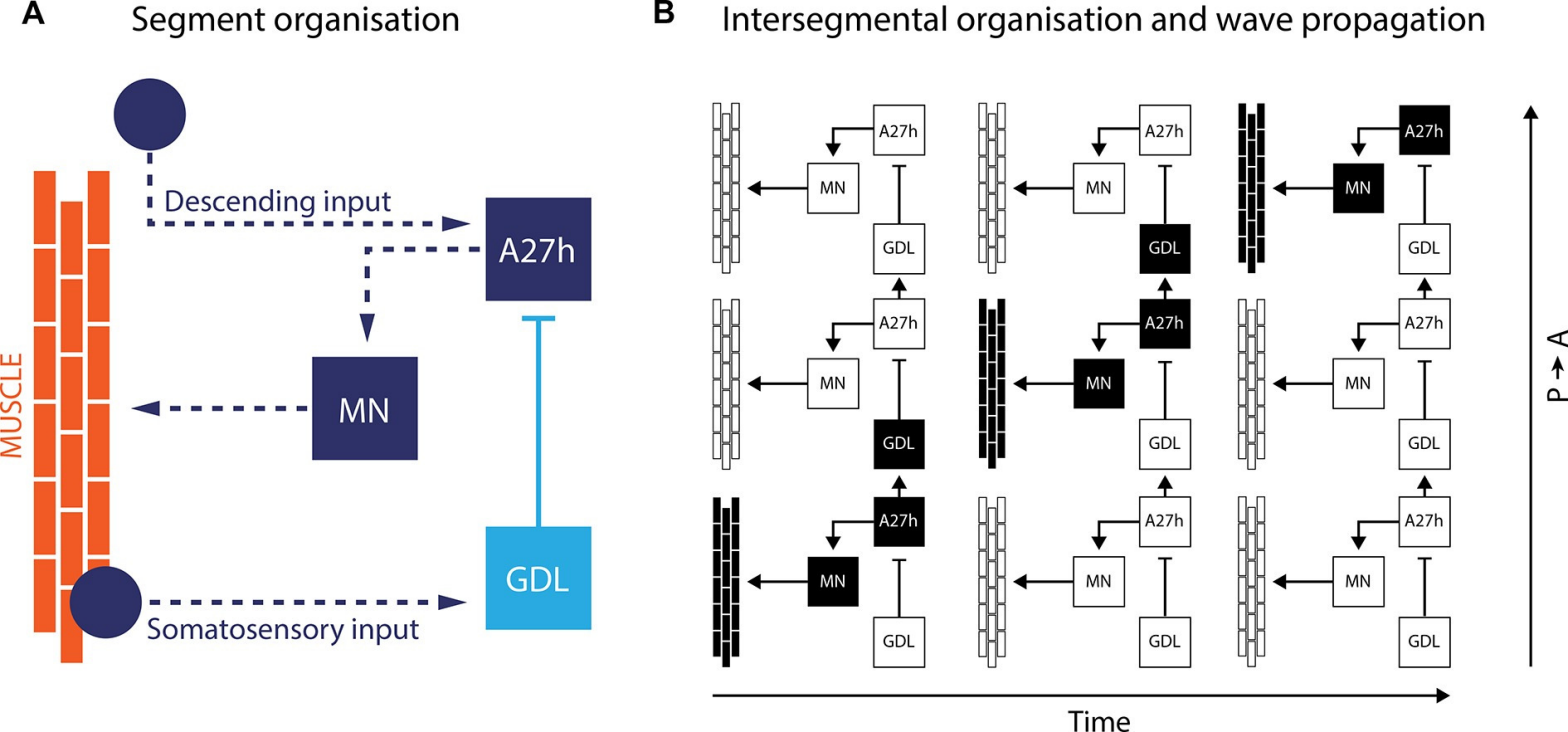
A neural circuit for crawling in fruit fly larvae. (**A**) The motor neurons (MN) in a given body segment trigger the contraction of abdominal muscles in that segment (orange). Two other neurons control each motor neuron: the A27h neuron (dark blue) activates the motor neuron, and the GDL neuron (light blue) inhibits the A27h neuron. The circuit formed by these three neurons also receives input from the brain (descending input), input from other segmental neurons (not shown) and feedback signals from muscle (somatosensory input). These three inputs are likely to modulate the speed of locomotion. (**B**) In order to generate a locomotion pattern such as crawling, it is essential to orchestrate the contraction of muscles along the body. During forward movement, the A27h neuron in a given segment is active and drives contraction of the corresponding muscles (black: active elements, white: inactive elements). It also activates the GDL neuron in the next segment, which inactivates the corresponding A27h neuron and prevents the muscles in this segment from contracting at the same time. Then, A27h activity in the first segment declines, which relieves the inhibition of A27h in the second segment and allows the muscles to contract. P → A: posterior (back) to anterior (front)

The isolated ventral nerve cords do not include any inputs from sensory neurons, and so the findings support the view that these neurons might indeed be part of a crawling central pattern generator that does not need sensory feedback to operate. However, Fushiki et al. also identified two types of somatosensory neurons that form contacts with A27h and GDL neurons. Therefore, it is possible that sensory input through this pathway is necessary to control how fast the larvae crawl.

So where does this study leave us? The neural circuit described by Fushiki et al. appears to be composed of a chain of strongly interconnected groups of neurons that resemble the nearest-neighbour-only design (Gjorgjieva et al., 2013). In this model, inhibitory coupling between neurons in neighbouring segments sets up the staggered pattern of motor neuron activity needed for the waves of muscle contraction. The GDL neuron seems to be at the core of this coupling. However, the extent to which the groups of neurons in individual segments are able to operate independently and still mantain a certain rhythmicity is not clear. Moreover, we do not know if all the individual micro-circuits along the body of the larvae oscillate with the same period, as this is not the case in other insects and their relatives.

This study shows how the extraordinary advancements in electron microscopy combined with better genetic tools have finally opened a door to studying neural circuits that were previously hard to reach. It would seem that the days when we used to happily roam the paths of the brain without a map are about to end. Mourn or rejoice, as you please.
